# How could preventive therapy affect the prevalence of drug resistance? Causes and consequences

**DOI:** 10.1098/rstb.2014.0306

**Published:** 2015-06-05

**Authors:** Amber Kunkel, Caroline Colijn, Marc Lipsitch, Ted Cohen

**Affiliations:** 1 Department of Epidemiology, Harvard T. H. Chan School of Public Health, Boston, MA 02115, USA; 2 Department of Mathematics, Imperial College, London SW7 2AZ, UK; 3 Department of Epidemiology of Microbial Diseases, Yale School of Public Health, New Haven, CT 06520, USA

**Keywords:** prophylaxis, preventive, mathematical model, antibiotic resistance, indirect effects, competition

## Abstract

Various forms of preventive and prophylactic antimicrobial therapies have been proposed to combat HIV (e.g. pre-exposure prophylaxis), tuberculosis (e.g. isoniazid preventive therapy) and malaria (e.g. intermittent preventive treatment). However, the potential population-level effects of preventative therapy (PT) on the prevalence of drug resistance are not well understood. PT can directly affect the rate at which resistance is acquired among those receiving PT. It can also indirectly affect resistance by altering the rate at which resistance is acquired through treatment for active disease and by modifying the level of competition between transmission of drug-resistant and drug-sensitive pathogens. We propose a general mathematical model to explore the ways in which PT can affect the long-term prevalence of drug resistance. Depending on the relative contributions of these three mechanisms, we find that increasing the level of coverage of PT may result in increases, decreases or non-monotonic changes in the overall prevalence of drug resistance. These results demonstrate the complexity of the relationship between PT and drug resistance in the population. Care should be taken when predicting population-level changes in drug resistance from small pilot studies of PT or estimates based solely on its direct effects.

## 1. Introduction

Preventive and prophylactic infectious disease therapies (we will refer to both collectively as preventive therapy, PT) involve the use of chemotherapeutic agents in asymptomatic and non-infectious individuals, with the goal of preventing future symptoms and infectiousness. PT may be applied to individuals who are either uninfected or latently infected with a given pathogen. For example, whereas isoniazid preventive therapy for tuberculosis (TB) can prevent disease progression in latently infected individuals [1,2], pre-exposure prophylaxis for human immunodeficiency virus (HIV) is intended solely for use in uninfected individuals [3]. Some interventions may include aspects of both treatment and PT; for example, intermittent preventive treatment for malaria involves a full course of antimalarial treatment applied irrespective of infection status [4].

Because PT prevents development of infectiousness as well as symptoms, PT has been proposed as an element of public health strategies aimed at reducing the burden of TB, HIV and malaria [4–6]. However, such strategies have often been controversial, with concerns about drug resistance forming one major barrier to implementation [7,8]. When the chemotherapeutic agents that are used for prevention are also needed for treatment, any drug resistance produced or amplified as a result of PT may undermine future control efforts. Simulation models intended to assess the potential effects of PT on the prevalence of drug resistance have produced sometimes inconsistent results [9]. For example, Supervie *et al.* [10,11] predicted that rolling out pre-exposure prophylaxis in Botswana would reduce the prevalence of drug-resistant (DR) HIV, whereas Abbas *et al.* [12,13] predicted that a similar programme in South Africa would increase the prevalence of DR HIV.

Models intended to predict the effects of specific PT programmes tend to be fairly complex, with states and parameters chosen to reflect the natural history of the disease of interest, the operational details of the proposed intervention and the efficacy of the available drug. While this complexity may improve the predictive accuracy of each individual model, it can complicate attempts to explain differences in their predictions [9,11,13]. In this paper, we create a simplified, general model of PT with the goal of better understanding the ways in which PT could alter the population prevalence of drug resistance. We show that increasing PT coverage can have qualitatively different effects on the prevalence of drug resistance depending on the relative importance of resistance acquired as a result of PT, resistance acquired as a result of treatment and the competitive fitness of DR strains.

## 2. Material and methods

We developed a simple mathematical model to demonstrate the ways in which PT may alter the prevalence of drug resistance. Mathematical modelling provides a way to formally encode our understanding of the individual-level effects of PT, some of which may lead to drug resistance. Furthermore, mathematical modelling creates a conceptual framework to explore how the effects of PT on drug resistance in the population may extend beyond its immediate recipients.

### (a) Model structure: disease course

**Figure 1.**
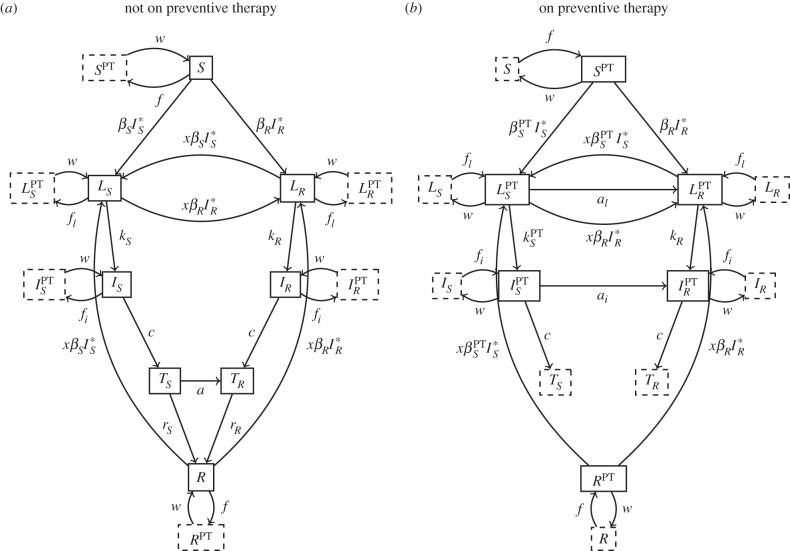
(*a*) All states and transitions involving individuals not on PT (solid boxes), with transitions on and off PT shown via links to on-PT states (dashed boxes). (*b*) All states and transitions involving individuals on PT (solid boxes), with transitions off and on PT shown via links to off-PT states (dashed boxes).

A description of the states and parameters used in our model is given in table 1. Figure 1 displays the structure of this compartmental model, with the health states and transitions among individuals not receiving PT on the left-hand side (*a*) and among individuals receiving PT on the right-hand side (*b*). We focus first on individuals not receiving PT (figure 1*a*). Although this portion of the figure shows the rates at which individuals may begin and end PT (PT states shown in dotted boxes), it does not display transitions between PT states.

**Table 1.** Model states and parameters.

state	name	description (all states: proportion of population)
*S*	susceptible	uninfected, negative infection history
*L_S_*	DS latent	latently infected with DS strain
*L_R_*	DR latent	latently infected with DR strain
*I_S_*	DS actively infected	infectious with DS strain, not on treatment
*I_R_*	DR actively infected	infectious with DR strain, not on treatment
*T_S_*	DS treated	infectious with DS strain, on treatment
*T_R_*	DR treated	infectious with DR strain, on treatment
I_S_*	total DS infectious	sum of DS infectious states: 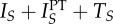
I_R_*	total DR infectious	sum of DR infectious states: 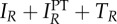
*R*	recovered	uninfected, positive infection history
parameter	name	description
*β_S_*	DS transmission parameter	# DS effective contacts per susceptible per unit time
*β_R_*	DR transmission parameter	# DR effective contacts per susceptible per unit time
*k_S_*	DS progression rate	rate of progression from DS latent to DS actively infected
*k_R_*	DR progression rate	rate of progression from DR latent to DR actively infected
*c*	case detection rate	rate at which actively infected individuals begin treatment
*r_S_*	DS recovery rate	rate of recovery from DS treated to recovered
*r_R_*	DR recovery rate	rate of recovery from DR treated to recovered
*a*	treated resistance rate	rate resistance is acquired due to treatment
*a_l_*	PT latent resistance rate	rate resistance is acquired by DS latents on PT
*a_i_*	PT active resistance rate	rate resistance is acquired by DS actively infecteds on PT
*x*	reinfection susceptibility	susceptibility retained after initial infection
*w*	PT exit rate	reciprocal of average duration of PT
*f*	PT uninfected start rate	start rate of PT for uninfected individuals
*f_l_*	PT latent start rate	start rate of PT for latently infected individuals
*f_i_*	PT active start rate	start rate of PT for actively infected individuals
superscript	name	description
PT	preventive therapy	state/parameter refers to individuals receiving PT

Within the model, an individual may be infected by pathogen phenotypes that are either drug sensitive (DS, indicated in the diagram by a subscript S) or DR (indicated in the diagram by a subscript R), but not by both simultaneously. Not allowing for mixed infections greatly simplifies our model, but introduces strong assumptions about competition between strains, the implications of which are considered in the Discussion. Susceptible (*S*) persons who are infected enter latency with either the DS strain (*L_S_*) or the DR strain (*L_R_*), depending on the source of the infection. Latently infected individuals may be superinfected and move to the latent state characterized by the drug sensitivity pattern of the most recently infecting strain. We assume the degree of susceptibility to reinfection *x* does not depend on the identity of the initial or reinfecting strain. We do allow the risks of infection and progression to active disease to differ based on the drug sensitivity of the infecting strain, reflecting the potential fitness costs of resistance.

All actively infected individuals within our model, including those on treatment, contribute to the overall force of infection. We assume that infectious individuals cannot be reinfected and cannot recover except by treatment. We allow individuals receiving treatment for DS disease to acquire resistance at rate *a*. We assume such acquired resistant cases are immediately detected and started on treatment for DR disease, which we assume has a lower cure rate than treatment for DS disease. We do not allow for disease-induced mortality or explicitly encode for treatment failure, though the latter may be incorporated into the treatment cure rate. Once cured, individuals revert to a recovered (*R*) state exhibiting the same level of immunity as that experienced by latently infected individuals.

Though we omit arrows representing mortality from figure 1, we assume a constant mortality rate from each compartment and a constant population size. All individuals enter the model susceptible to infection and not on PT. Because we assume a fixed population size, we express all states in terms of proportion of the population.

### (b) Model structure: preventive therapy

Figure 1*b* displays the portion of our model pertaining to individuals receiving PT. This portion of the figure again displays the rates at which individuals may begin or end PT (non-PT states shown in dotted boxes), but omits arrows indicating the transitions between states of individuals not receiving PT. We allow for individuals who are uninfected, latently infected or actively infected to potentially receive PT. Uninfected individuals begin PT at rate *f* and cease therapy at rate *w*. Latently infected individuals begin PT at rate *f_l_* and cease therapy at rate *w*. We allow the rates at which uninfected and latently infected individuals initiate PT to differ, as the specific targeting of PT depends on the disease and drug of interest. Pre-exposure prophylaxis for HIV, for example, is intended solely for uninfected individuals [3], whereas isoniazid preventive therapy is typically targeted to individuals with latent TB infection [1,2]. We assume that the PT initiation rate is the same for both DS and DR latently infected individuals, assuming that the resistance phenotype of the infecting strain is not known during latency. Actively infected individuals may also receive PT within our model. Though PT is generally not intended for such individuals (except when the same drug is applied as both treatment and prevention, e.g. intermittent preventive treatment for malaria [4]), individuals may progress from latent to active infection while receiving PT (rate 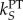) or initiate PT during active disease as a result of imperfect screening (rate *f_i_*). We assume that the PT start rate is the same for both DS and DR actively infected individuals, assuming the infection is not recognized prior to PT initiation. We assume that actively infected individuals cease PT routinely, at rate *w*, or upon initiation of treatment, at the same case detection rate *c* as for individuals not receiving PT.

The health states for individuals receiving PT are similar to those described for individuals not receiving PT. We assume PT reduces the rate at which uninfected and latently infected individuals are infected with the DS strain (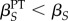), the rate at which DS latently infected individuals progress to active disease (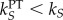), or the rates of both infection and progression with the DS strain. Although we assume that PT has no direct effect on infection or progression with the DR strain, it may affect the probability of progression with the DR strain by changing the probability of reinfection with the DS strain. We allow DS latently infected individuals to acquire resistance as a result of PT at rate *a_l_* and DS actively infected individuals at rate *a_i_*. We assume PT does not cure or reduce the infectiousness of individuals with active infection. We also assume that individuals cannot receive PT and treatment simultaneously, but treated individuals again become eligible for PT upon recovery. Throughout our analysis, we do not track which individuals receive PT and thus assume that the same individuals may receive multiple courses of PT.

### (c) Outcome measures

Throughout our analysis, we focus on the equilibrium behaviour of the model. Doing so simplifies our analysis by removing its dependence on the initial model conditions. We begin each of our analyses in the absence of PT (setting the PT start rates *f* = *f_l_* = *f_i_* = 0). For each of our analyses, we choose a parameter set such that, in the absence of PT, the equilibrium prevalence of the DS strain is non-zero and the basic reproductive number of the DR strain exceeds 1. Because we allow for acquired resistance, the former requirement implies that the equilibrium prevalence of the DR strain is also non-zero in the absence of PT (i.e. there is no DS only equilibrium). The latter implies that the equilibrium prevalence of the DR strain will remain non-zero even if the equilibrium prevalence of the DS strain does not.

Holding this parameter set fixed, including the rates of case detection and treatment for active disease, we run a series of simulations at progressively higher values of the PT initiation rate. For the purpose of our simulations, we assume the PT start rates among uninfected, latently infected and infectious individuals are proportional throughout, with *f_l_* = *f* and *f_i_* = *f*/10, and thus refer to the PT start rate using the single parameter *f*. For each individual simulation, we fix the value of the PT initiation rate, run the model to equilibrium (i.e. until changes in population composition between time steps become negligible), and record the resulting prevalence of the DR strain. We repeat the simulation process for incrementally increasing values of *f* until the DS strain is eliminated (the equilibrium prevalence of the DS strain equals 0), still holding the PT initiation rate constant within each individual simulation. Because we do not allow DR strains to revert to DS, such elimination of the DS strain is possible in our model even when the equilibrium prevalence of the DR strain remains non-zero.

All of the results provided are based on model simulations created using the R differential equation solver ‘ode’ within package deSolve.

## 3. Results

In our model, increasing the intensity of PT directly affects the amount of resistance acquired through PT. It also indirectly affects the amount of resistance acquired through treatment for active disease and the competitive transmission advantage afforded to DR strains. We find that the combined effects of these mechanisms can result in increasing, decreasing, and non-monotonic relationships between the intensity of PT coverage and DR prevalence. Throughout the results, we use the word ‘treatment’ to refer solely to treatment for active disease.

### (a) Preventive therapy coverage and resistance acquired through preventive therapy

**Figure 2.**
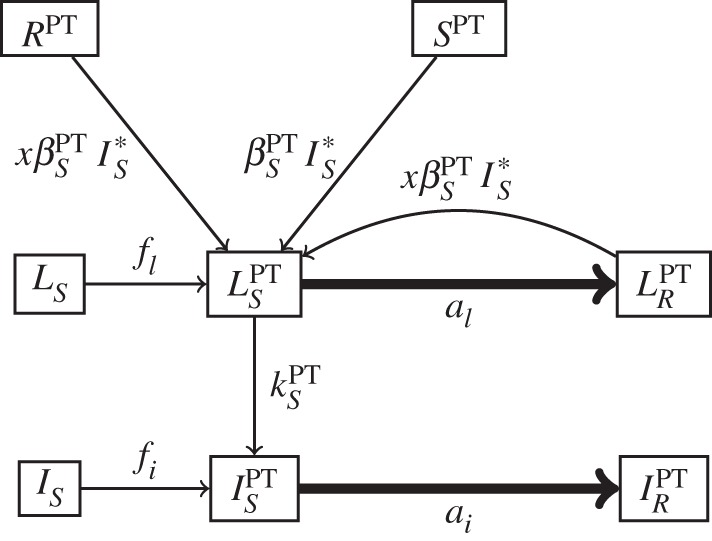
Subset of the model representing the rates at which individuals with latent or active DS disease receiving PT (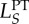 and 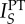, respectively) acquire resistance (bold) and the transitions leading to these potentially at-risk states.

In our model, PT may lead directly to acquired resistance among individuals latently or actively infected with the DS strain. To demonstrate how it may do so, figure 2 provides a focused view of the relevant states and transitions from figure 1. Unbolded arrows in figure 2 show the transitions that may lead to individuals latently or actively infected with the DS strain receiving PT. Bolded arrows show the acquisition of resistance among such individuals as a result of PT. If no individuals are to acquire resistance as a result of PT, one of the following scenarios must apply: (i) no individuals with active or latent infection ever receive PT, (ii) no individuals with active infection ever receive PT, and PT never results in acquired resistance among latently infected individuals or (iii) PT never results in acquired resistance among latently or actively infected individuals. The first scenario assumes that PT is intended only for uninfected individuals, that screening for latent and active infection prior to PT initiation is perfect (*f_i_* = 0 and *f_l_* = 0) and that adherence and drug efficacy are sufficiently high that individuals receiving PT never become infected (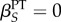). The second scenario assumes that PT never selects for sporadically occurring resistant mutants among individuals with latent infection (*a_l_* = 0), that screening for active infection prior to PT initiation is perfect (*f_i_* = 0), and that adherence and drug efficacy are sufficiently high that individuals receiving PT never progress from latent to active infection (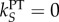). The third scenario assumes that PT is incapable of selecting for resistance at the individual level among both latently and actively infected individuals (*a_l_* = 0 and *a_i_* = 0). Even well-functioning PT programmes are unlikely to meet these stringent criteria, and thus it is reasonable to expect that some individuals will directly acquire resistance as a result of PT.

**Figure 3.**
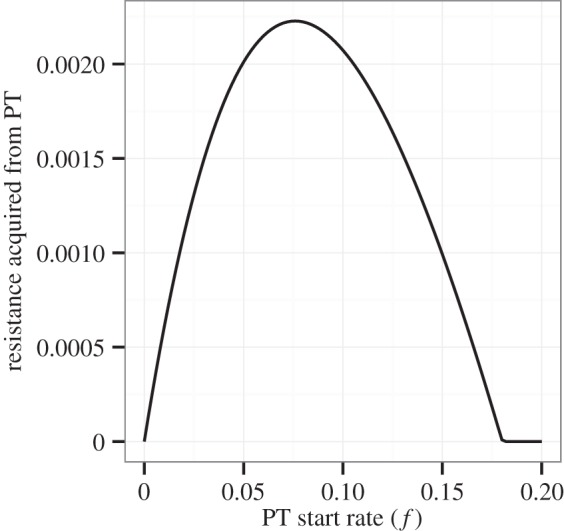
The relationship between PT start rate *f* and the rate at which resistance is acquired through PT (
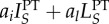
) at equilibrium. Parameters for this figure: *μ* = 0.02, *r_R_* = 1, *r_S_* = 2, *c* = 1, *k_R_* = 1, *k_S_* = 1.5, *β_S_* = 2, *β_R_* = 1, *x* = 1, *a* = 0.3, *a_i_* = 0.5, *a_l_* = 0.1, *w* = 0.1, 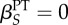, 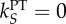.

When we assume that some or all of these parameters are non-zero, reflecting the vast majority of real-world PT applications, the relationship between PT coverage and resistance acquired as a result of PT is shown in figure 3. The level of resistance acquired through PT is a function of the number of DS actively and latently infected individuals receiving PT (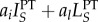). When PT coverage is low and insufficiently able to control the epidemic, increasing PT coverage increases the number of latently and actively infected individuals receiving PT and thus the number of people who acquire resistance as a result of PT. When PT coverage is high and better able to control the epidemic, increasing PT coverage decreases the number of people who acquire resistance as a result of PT (similar to an effect described in [14]). Under such scenarios, although increasing the PT initiation rate still increases the total number of people receiving PT, the resulting reduction in the force of DS infection is sufficient to decrease the number of people receiving PT who have latent or active DS infection. Because only DS infected individuals are at risk of acquiring resistance as a result of PT, this results in a reduction of the rate at which resistance is acquired as a result of PT.

### (b) Preventive therapy coverage and resistance acquired through treatment

**Figure 4.**
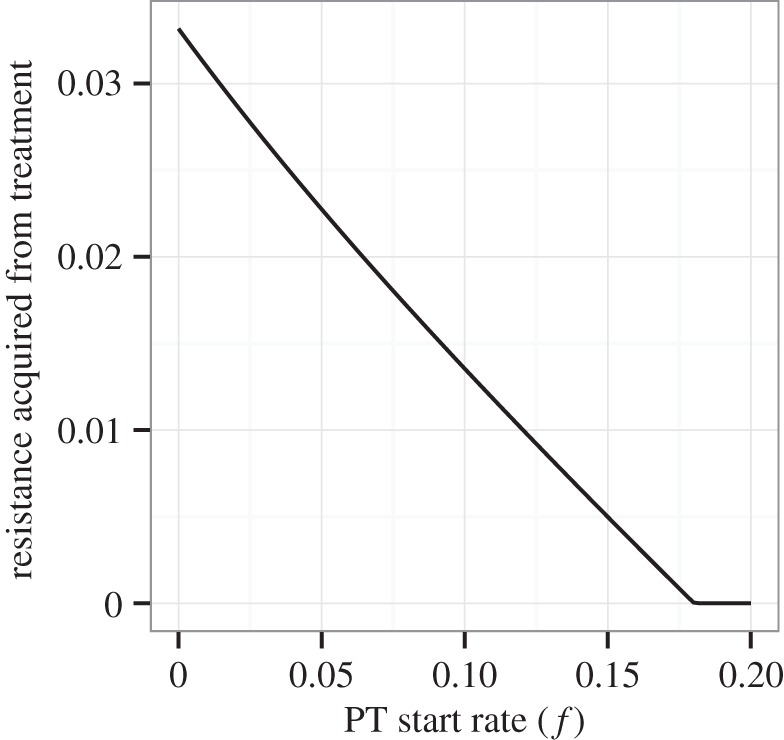
The relationship between PT start rate *f* and the rate at which resistance is acquired through treatment for DS disease (*aT_S_*) at equilibrium. Parameters for this figure are the same as those for figure 3.

As shown in figure 1, our model allows individuals receiving treatment for active DS disease (*T_S_*) to acquire resistance at rate *a*. Increasing the coverage of PT in the population decreases the number of people infected with the DS strain, and thus decreases the number of people who acquire resistance through treatment for active disease. This relationship is shown in figure 4.

### (c) Preventive therapy coverage and transmission of the drug-resistant strain

Our model assumes high levels of competition for susceptible hosts between strains, as we do not allow for latent or active co-infection. As a result, increasing PT coverage may provide a selective advantage to DR strains through two distinct mechanisms. First, increasing PT coverage increases the probability that an individual latently infected with the DR strain will progress to active DR infection. This relationship is a result of our assumption that DR latently infected individuals could potentially be ‘rescued’ from progressing to DR disease by superinfection with the DS strain. As PT coverage increases, DR latently infected individuals are increasingly protected from such superinfection and are therefore more likely to progress with their DR strain. Second, increasing PT coverage increases the proportion of DR uninfected individuals who are susceptible to the DR strain. In our model, the proportion of all individuals who are susceptible to the DR strain is given by *S* + *xL_S_* + *xR* + *S*^PT^ + *xL_S_*^PT^ + *xR*^PT^, which depends on the proportion of people uninfected by the DR strain, the proportion of people with active DS infection, and the level of immunity afforded by initial infection. To obtain the proportion of DR uninfected individuals who are susceptible to DR infection, we divide this by the total proportion of individuals not actively or latently infected with the DR strain (*S* + *R* + *L_S_* + *S^PT^* + *R*^PT^ + *L_S_*^PT^ + *I_S_* + *I_S_*^PT^ + *T_S_*). Increasing PT coverage reduces the number of persons with active DS infection, and therefore increases the proportion of DR uninfected individuals who are susceptible to the DR strain. These two effects are discussed in more detail in the electronic supplementary material.

The effective reproductive number of the DR strain is a composite measure that allows us to assess the combined effects of these mechanisms on the competitive fitness of the DR strain. The effective reproductive number shows the number of secondary infectious cases produced by a single infectious individual over the course of their infectious period. As opposed to the basic reproductive number *R*_0_, which assumes a wholly susceptible population, the effective reproductive number at a given time point depends on the susceptibility pattern of the population at that point in time. In a single strain model, the effective reproductive number at equilibrium is equal to 1. In our model, however, the number of DR infected individuals is boosted by acquired resistance, and therefore the DR strain may coexist with the DS strain in the population even when the effective reproductive number of the DR strain is below 1.

**Figure 5.**
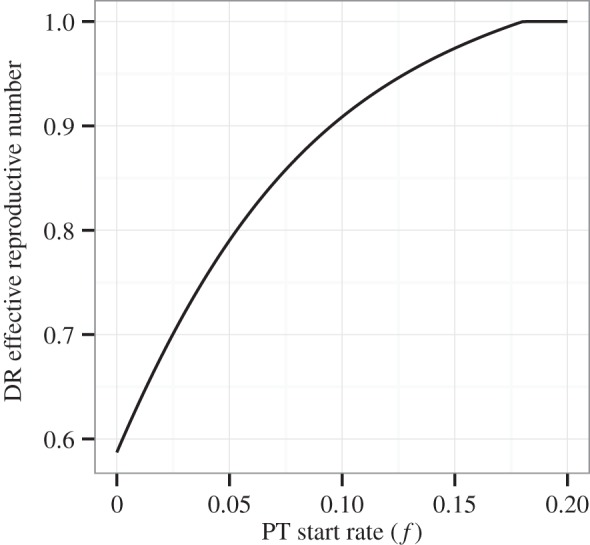
The relationship between PT start rate *f* and the effective reproductive number of the DR strain at equilibrium. Calculations are given in the electronic supplementary material. Parameters for this figure are the same as those for figure 3.

Figure 5 shows how the effective reproductive number of the DR strain at equilibrium changes as PT coverage increases. At low PT coverage levels, the DR effective reproductive number is less than 1, indicating that acquired resistance is necessary for the persistence of the DR strain in the population. As PT coverage increases, the reproductive fitness of the DR strain increases as well. When PT coverage is sufficiently high, the DR effective reproductive number reaches 1, indicating that resistance has become self-sustaining and the DR strain has overtaken the DS strain in the population.

### (d) Composite effects of preventive therapy coverage on drug-resistant prevalence

**Figure 6.**
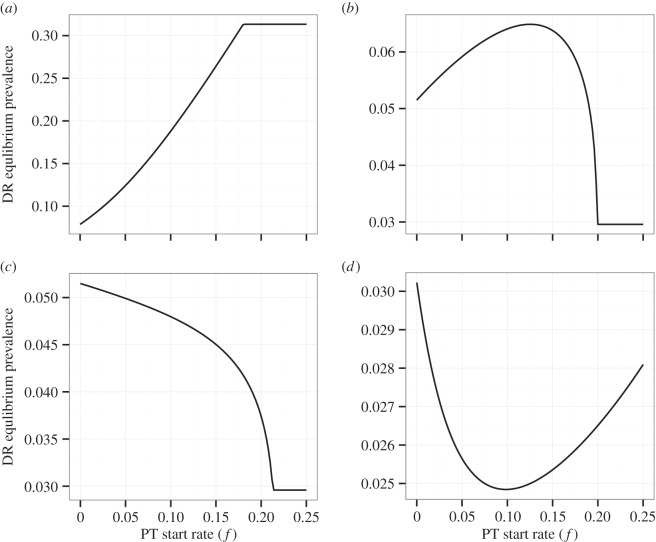
Relationship between PT start rate *f* and DR prevalence (
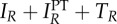
) at equilibrium. Parameters for (*a*) are the same as those from figures 3–5: *μ* = 0.02, *r_R_* = 1, *r_S_* = 2, *c* = 1, *k_R_* = 1, *k_S_* = 1.5, *β_S_* = 2, *β_R_* = 1, *x* = 1, *a* = 0.3, *a_i_* = 0.5, *a_l_* = 0.1, *w* = 0.1, 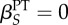, 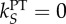. Parameters for (*b*): same as for (*a*), except *β_R_* = 0.55. Parameters for (*c*): same as for (*a*), except *β_R_* = 0.55, *a_i_* = 0, *a_l_* = 0. Parameters for (*d*): same as for (*a*), except *x* = 0.4, *a_i_* = 0, *a_l_* = 0, 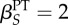. The same range of PT start rates is shown for each subplot, though this range is insufficient to cause elimination of the DS strain in (*d*).

Table 2 summarizes the ways in which each of the resistance mechanisms outlined above will tend to alter DR prevalence. While increasing PT coverage can decrease the rate of resistance acquired due to treatment, it can also increase the competitive transmission advantage of circulating DR strains, and its effects on the rate of resistance acquired due to PT are non-monotonic. Furthermore, in our model as in reality, none of these mechanisms exist in isolation. Rather, increasing PT coverage acts simultaneously on the rate at which resistance is acquired through PT, the rate at which resistance is acquired through treatment, and the competitive fitness of the DR strain. In figure 6, we show that the interactions between these mechanisms are sufficient to produce a range of qualitatively distinct relationships between PT coverage and equilibrium DR prevalence. Though the behaviours shown in this figure occur with varying frequencies and are not necessarily exhaustive, they demonstrate the complexity of the changes in DR prevalence that may result from PT.

**Table 2.** Summary of mechanisms through which PT may affect the prevalence of drug resistance. The proportion susceptible to the DR strain and the reproductive number of the DR strain are discussed in more detail in the electronic supplementary material.

	influence driven by		effect on DR prevalence for	
source of resistance	health states	parameters	low PT coverage	high PT coverage
PT	DS infected on PT ( 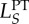 , 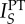 )	rate resistance acquired on PT (*a_l_*, *a_i_*)	↑	↓
treatment	DS treated (*T_S_*)	rate resistance acquired on treatment (*a*)	↓	↓
transmission	susceptible to DR strain	reproductive number of DR strain	↑	↑

Figure 6*a*–*d* was created using the same model of PT under different sets of parameters. The parameters used for each subplot are shown in the figure caption. In figure 6*a*, DR prevalence increases monotonically with PT coverage. The parameters used to produce this subplot were the same as those used to create the figures for the previous sections. In figure 6*b*, DR prevalence increases with PT coverage when PT coverage is low, but decreases with increasing PT coverage if PT coverage exceeds a threshold value. To create this subplot, we lowered the transmission parameter for the DR strain *β_R_*. This decrease in the transmissibility of the DR strain allows acquisition of resistance through PT and treatment to play a larger role in changing DR prevalence. In figure 6*c*, DR prevalence decreases monotonically with increasing PT coverage. To create this subplot, we lowered the transmission parameter of the DR strain as in (*b*) and assumed that no resistance was acquired as a result of PT, allowing acquisition of resistance by treatment alone to become the major driver of DR prevalence. Finally, in figure 6*d*, DR prevalence decreases with increasing PT coverage when PT coverage is low, but increases with increasing PT coverage if PT coverage exceeds a threshold value. To create this subplot, we lowered the reinfection susceptibility of latently infected and recovered individuals, assumed no resistance acquired as a result of PT, and assumed PT did not affect infection with the DS strain (i.e. that it only affected disease progression). The resulting U-shaped curve indicates that, at low coverage levels, PT primarily influences resistance acquired due to treatment for active disease, whereas at high coverage levels, PT exerts more influence by allowing greater transmission of the DR strain. This relationship may reflect the fact that lowering the progression rate affects the prevalence of latent DS infection differently than the rate of active DS infection, complicating the association between the prevalence of DS disease and the number of people susceptible to infection with the DR strain. Note that the absolute changes in DR prevalence in this subplot are small; nevertheless, this shape further reflects the complexity of the ways in which PT may cause changes in DR prevalence.

## 4. Discussion

Mathematical models of varying complexity have been constructed to predict the effects of pre-exposure prophylaxis for HIV [10,12,15], isoniazid preventive therapy for TB [16–18] and intermittent preventive treatment for malaria [19,20] on the prevalence of drug resistance. Here, we have used a more general model to provide an overall view of the ways in which PT may influence the prevalence of drug resistance and the anticipated directions of these effects.

First, we have described the relationship between PT coverage and the amount of resistance acquired directly as a result of PT. Previous models have demonstrated particular sensitivity to assumptions surrounding the use of PT in infected individuals [21]. Our model shows that when PT coverage is low, increasing PT coverage increases the amount of resistance acquired as a result of PT. When PT coverage is high, however, further increasing PT coverage decreases the amount of resistance acquired as a result of PT, resulting in an inverted U-shaped curve between PT coverage and resistance acquired from PT. A similar relationship has been described between drug pressure and the rate of resistance in the setting of treatment for active disease [14]. Notably, this resistance mechanism is not a necessary consequence of the beneficial effects of PT. The number of people who acquire resistance as a result of PT may be reduced by limiting the number of infected individuals started on PT (e.g. through better screening programmes), the number of individuals receiving PT who develop latent or active infection (e.g. through better adherence or more effective PT drugs), and the rate at which infected individuals on PT acquire resistance (e.g. through drugs or drug combinations more similar to those used for treatment).

Second, we have shown that increasing PT coverage decreases the amount of resistance acquired as a result of treatment for active disease. This relationship occurs because PT decreases the number of individuals with active DS disease. We would expect a similar relationship to hold for non-therapeutic interventions that do not exclusively target DS disease, such as condom use in the setting of HIV.

Third, we have demonstrated that increasing PT coverage provides a selective advantage to circulating DR strains. We have found that increasing PT coverage increases the effective reproductive number of the DR strain, which is consistent with predictions and observations for vaccines targeting specific disease strains [22,23] and previous PT modelling papers that have used strain competition to explain predicted increases in DR prevalence [17,18]. Increasing the intensity of PT coverage increases the effective reproductive number of the DR strain by increasing the probability that a DR latently infected individual will progress to active DR infection (before reinfection with the DS strain) and by increasing the proportion of the DR uninfected population that is susceptible to infection with the DR strain.

Finally, we have shown that PT may have a wide range of effects on overall DR prevalence, depending on the interaction of these three mechanisms. Specifically, we have provided examples of increasing, decreasing, U-shaped and inverted U-shaped relationships between PT intensity and equilibrium DR prevalence resulting from our model. These four shapes are not necessarily exhaustive, but demonstrate that the relationship between PT coverage and DR prevalence may differ qualitatively depending on the disease and drug in question. In particular, predictions of the effects of PT on drug resistance are sensitive to a number of properties of the system: the rate at which resistance is acquired as a result of PT, the rate at which resistance is acquired as a result of treatment, the fitness costs of resistance on disease transmissibility, the mechanisms of PT and the rate of reinfection. Reliable estimates of these parameters are needed to accurately predict the effects of proposed PT programmes on DR prevalence. Our estimates are also sensitive to the assumption that individuals cannot be reinfected throughout their infectious periods, illustrating the importance of understanding within-host strain competition when predicting the population-level effects of PT.

Understanding how each of these factors contribute to the relationship between PT and drug resistance may aid in the interpretation of models with differing predictions about the effects of PT on drug resistance. For example, our analysis sheds some additional light on the observations made by Abbas *et al.* [13] on the sources of difference in the model predictions of Supervie *et al.* [10] and Abbas *et al.* [12]. Abbas *et al.* [13] re-created both models to explore the reasons for contrasting conclusions about the potential relationship between pre-exposure prophylaxis (PrEP) and HIV drug resistance in sub-Saharan Africa. They suggest that a low value of *R*_0_ contributed to PrEP decreasing the prevalence of drug resistance in Supervie *et al*. [10], which accords with our demonstration that although PT provides a competitive advantage to DR strains, it may still reduce the overall prevalence of drug resistance when the transmissibility of the DR strain is low and resistance is driven primarily by acquisition. Similarly, their observation that the differences between the two models could be partially explained by differing PrEP coverage rates is supported by our finding that the effects of increasing PT coverage may be non-monotonic. The authors also acknowledge that resistance in the population occurs as a result of transmission and treatment (i.e. antiretroviral therapy) as well as PrEP; as we have shown, the effects of PT on drug resistance cannot be distilled to its effects on resistance acquired through PT alone.

We have presented a general model that may not perfectly reflect the natural history of any particular infection. Though in reality the specific action and targeting of PT varies depending on the disease and drug of interest, we assume PT protects both susceptible and latently infected individuals from active DS disease. Our assumption of no latent or active mixed infections encodes a high level of competition between strains for susceptible hosts, the biological plausibility of which will depend on the disease of interest. Other models have demonstrated that allowing for mixed infections may either heighten or mitigate the effective degree of competition between strains depending on assumptions of how strains compete within and between hosts [24–27]. If we could assume DS and DR strains are perfectly non-competing, changing PT coverage may not affect the effective reproductive number of the DR strain; however, we expect most pathogens to exhibit some level of competition between strains and therefore qualitative behaviours similar to those described here. In addition to the assumption of no mixed infections, we assume a binary designation of drug resistance that may not accurately represent the accumulation of resistance mutations within a single host. Furthermore, we do not allow DR strains to revert to DS, though this behaviour has been demonstrated for pathogens including HIV [28]. We assume that the effects of PT on disease progression cease immediately after PT is removed, and do not allow PT to increase the cure rate or reduce the infectiousness of infectious individuals (as might occur if the drugs used for PT are similar to those used for treatment). Similarly, we assume that PT has no direct effects on immunity to future infection. Finally, we focus our analysis on the effects of PT on drug resistance at equilibrium, even though policy-makers may be most interested in its short-term effects.

Nevertheless, we have provided a systematic account of both direct and indirect mechanisms through which PT may affect DR prevalence. Depending on the relative contributions of these resistance mechanisms, raising PT coverage could have increasing, decreasing or non-monotonic effects on long-term DR prevalence. Because these relationships may be non-monotonic, care should be taken when extrapolating the effects of small PT programmes to larger efforts.

## Supplementary Material

Supplementary Material
