# Supplementary material for: How could preventive therapy affect the prevalence of drug resistance? Causes and consequences
Source: Philos Trans R Soc Lond B Biol Sci. 2015 Jun 5;370(1670):20140306. doi: 10.1098/rstb.2014.0306 (PMC4424438; doi:10.1098/rstb.2014.0306)
Supplement: Supplementary Material [file rstb20140306supp1.pdf]

Supplemental Material

Amber Kunkel, Caroline Colijn, Marc Lipsitch, Ted Cohen 2015 How could preventive therapy affect the prevalence of drug resistance? Causes and consequences. *Phil. Trans. R. Soc. B.* doi: 10.1098/rstb.2014.0306

A Equations

The states and parameters used here are the same as those described in Table 1 in the main text.

$$
\begin{aligned}
\dot{S}^{PT} &= fS - wS^{PT} - \beta_S^{PT}(I_S + I_S^{PT} + T_S)S^{PT} - \beta_R(I_R + I_R^{PT} + T_R)S^{PT} - \mu S^{PT} \\
\dot{L_S} &= \beta_S(I_S + I_S^{PT} + T_S)(S + xL_R + xR) - x\beta_R(I_R + I_R^{PT} + T_R)L_S - (k_S + \mu + f_l)L_S + wL_S^{PT} \\
\dot{L_R} &= \beta_R(I_R + I_R^{PT} + T_R)(S + xL_S + xR) - x\beta_S(I_S + I_S^{PT} + T_S)L_R - (k_R + \mu + f_l)L_R + wL_R^{PT} \\
\dot{L}_S^{PT} &= \beta_S^{PT}(I_S + I_S^{PT} + T_S)(S^{PT} + xL_R^{PT} + xR^{PT}) - x\beta_R(I_R + I_R^{PT} + T_R)L_S^{PT} \\
&\quad - (k_S^{PT} + \mu + w + a_l)L_S^{PT} + f_lL_S \\
\dot{L}_R^{PT} &= \beta_R(I_R + I_R^{PT} + T_R)(S^{PT} + xL_S^{PT} + xR^{PT}) - x\beta_S^{PT}(I_S + I_S^{PT} + T_S)L_R^{PT} \\
&\quad - (k_R + \mu + w)L_R^{PT} + f_lL_R + a_lL_S^{PT} \\
\dot{I_S} &= k_SL_S - (c + \mu + f_i)I_S + wI_S^{PT} \\
\dot{I_R} &= k_RL_R - (c + \mu + f_i)I_R + wI_R^{PT} \\
\dot{I_S^{PT}} &= k_S^{PT}L_S^{PT} - (c + \mu + a_i + w)I_S^{PT} + f_iI_S \\
\dot{I_R^{PT}} &= k_RL_R^{PT} + a_iI_S^{PT} - (c + \mu + w)I_R^{PT} + f_iI_R \\
\dot{T_S} &= c(I_S + I_S^{PT}) - (r_S + \mu + a)T_S \\
\dot{T_R} &= c(I_R + I_R^{PT}) - (r_R + \mu)T_R + aT_S \\
\dot{R} &= wR^{PT} - fR + r_ST_S + r_RT_R - x\beta_S(I_S + I_S^{PT} + T_S)R - x\beta_R(I_R + I_R^{PT} + T_R)R - \mu R \\
\dot{R}^{PT} &= fR - wR^{PT} - x\beta_S^{PT}(I_S + I_S^{PT} + T_S)R^{PT} - x\beta_R(I_R + I_R^{PT} + T_R)R^{PT} - \mu R^{PT} \\
S &= 1 - T_S - T_R - I_S - I_R - I_S^{PT} - I_R^{PT} - L_S - L_R - L_S^{PT} - L_R^{PT} - R - R^{PT} - S^{PT}
\end{aligned}
$$

B Calculating DR Effective Reproductive Number

This section refers to states and parameters described in Table 1 in the main text. The effective reproductive number is the number of secondary infectious cases produced by a single infectious individual over the course of their infectious period. We derived the effective reproductive number of the DR strain R_{RE} at equilibrium from first principles using the following equation:

$$R_{RE} = \beta_R D(P^0\theta_R^0 + P^{PT}\theta_R^{PT}).$$

We walk through each of the individual components of this equation below. β_R is the transmission parameter for the DR strain, as described in the main text.

D, the average duration of infectiousness with the DR strain, is the sum of two terms: 1) the average length of stay in the untreated infectious compartment and 2) the average length of stay in the treated infectious compartment given that the individual initiates treatment prior to death. This expression is given below:

$$D = \frac{1}{c+\mu} + \left(\frac{c}{c+\mu}\right)\left(\frac{1}{\mu + r_R}\right).$$

P^0, the probability of progressing from latent to active disease for individuals not on preventive therapy at the time of infection, is the sum of the probability of progressing before leaving L_R, the probability of starting preventive therapy and then progressing before leaving L_R^{PT}, the probability of starting preventive therapy and then stopping preventive therapy and then progressing before leaving L_R, and so on:

$$P^0 = \frac{k_R}{D_0} + \frac{f_l}{D_0}\frac{k_R}{D_{PT}} + \frac{f_l}{D_0}\frac{w}{D_{PT}}\frac{k_R}{D_0} + \frac{f_l}{D_0}\frac{w}{D_{PT}}\frac{f_l}{D_0}\frac{k_R}{D_{PT}} + ...$$

$$P^0 = \frac{k_R}{D_0}\sum_{i=0}^{\infty}\left(\frac{f_l}{D_0}\frac{w}{D_{PT}}\right)^i + \frac{f_l}{D_0}\frac{k_R}{D_{PT}}\sum_{j=0}^{\infty}\left(\frac{f_l}{D_0}\frac{w}{D_{PT}}\right)^j$$

where D_0 is the rate of exit from L_R

$$D_0 = k_R + \mu + \beta_S(I_S + I_S^{PT} + T_S) + f_l$$

and D_{PT} is the rate of exit from L_R^{PT}

$$D_{PT} = k_R + \mu + \beta_S^{PT}(I_S + I_S^{PT} + T_S) + w.$$

This expression for P^0 captures all of the possible paths from the latent state to the infectious state and hence captures the total probability of progression from latency to active DR disease. If we let $z = wf_l/(D_0 D_{PT})$ then the expression for P^0 simplifies to

$$P^0 = \frac{k_R}{D_0}\left(1 + \frac{z}{1-z}\right) + \frac{f_l}{D_0}\frac{k_R}{D_{PT}}\left(1 + \frac{z}{1-z}\right).$$

We can similarly derive the expression for P^{PT}, the probability of progressing from latent to active disease for individuals on preventive therapy at the time of infection, which simplifies to

$$P^{PT} = \frac{k_R}{D_{PT}}\left(1 + \frac{z}{1-z}\right) + \frac{w}{D_{PT}}\frac{k_R}{D_0}\left(1 + \frac{z}{1-z}\right).$$

Finally, θ_R^0 is the fraction of individuals who are susceptible to infection with the DR strain and not currently on PT:

$$\theta_R^0 = S + xR + xL_S$$

and θ_R^{PT} is the fraction of individuals on PT who are susceptible to infection with the DR strain:

$$\theta_R^{PT} = S^{PT} + xR^{PT} + xL_S^{PT}.$$

Individuals already infected with the DR strain are not included here, even though they may be reinfected with the DR strain, because they do not change states upon reinfection.

C DR Effective Reproductive Number Components

Changing the coverage of preventive therapy changes the DR effective reproductive number in two ways: by affecting the proportion of people infected with the DR strain who progress to active DR disease, and by affecting the proportion of the population that is susceptible to the DR strain. Here we show how each of these components are affected by changing PT coverage, using notation defined earlier in the appendix and in Table 1 in the main text.

The proportion of people infected with the DR strain who progress to active DR infection depends on the DS infection rate, which itself depends on the proportion of the population receiving preventive therapy. To produce a population average, we used the formula

$$P = \frac{P^0 \theta_R^0 + P^{PT} \theta_R^{PT}}{\theta_R^0 + \theta_R^{PT}}.$$

The results are shown in Supplemental Fig 1. The proportion of DR infected persons who progress to active infection with the DR strain increases with increasing PT coverage.

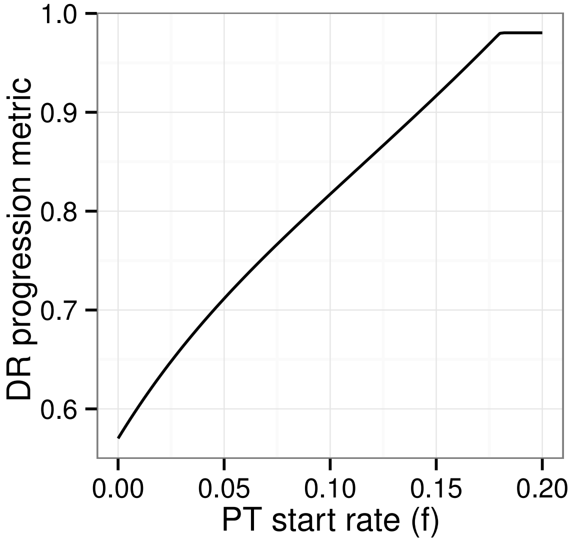

Figure 1: The relationship between PT start rate f and the proportion of people infected with the DR strain who progress to active DR infection. Parameters for this figure are the same as those for Fig 3 in the main text.

The proportion of people susceptible to the DR strain depends on the number of people uninfected with the DR strain without active DS infection and the level of immunity afforded by initial infection. To remove the effects of changing DR prevalence, we show here the proportion of DR uninfected persons who are susceptible to the DR strain:

$$\theta_R = \frac{\theta_R^0 + \theta_R^{PT}}{S + R + L_S + S^{PT} + R^{PT} + L_S^{PT} + I_S + I_S^{PT} + T_S}.$$

The results are shown in Supplemental Fig 2. The proportion of DR uninfected individuals who are susceptible to the DR strain increases with increasing PT coverage.

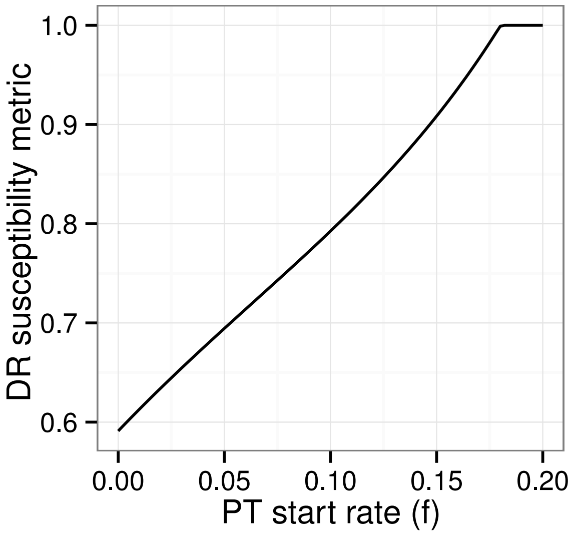

Figure 2: The relationship between PT start rate f and proportion of DR uninfected persons susceptible to the DR strain. Parameters for this figure are the same as those for Fig 3 in the main text.
